# Artificial neural network inference analysis identified novel genes and gene interactions associated with skeletal muscle aging

**DOI:** 10.1002/jcsm.13562

**Published:** 2024-08-29

**Authors:** Janelle Tarum, Graham Ball, Thomas Gustafsson, Mikael Altun, Lívia Santos

**Affiliations:** ^1^ Department of Sport Science, Sport, Health and Performance Enhancement Research Centre (SHAPE), School of Science and Technology Nottingham Trent University Nottingham UK; ^2^ Medical Technology Research Centre Anglia Ruskin University Essex UK; ^3^ Department of Laboratory Medicine, Section of Clinical Physiology Karolinska Institutet Huddinge Huddinge Sweden; ^4^ Department of Clinical Physiology Karolinska University Hospital Huddinge Sweden

**Keywords:** Aging, Artificial neural network, Exercise, Machine learning, Skeletal muscle

## Abstract

**Background:**

Sarcopenia is an age‐related muscle disease that increases the risk of falls, disabilities, and death. It is associated with increased muscle protein degradation driven by molecular signalling pathways including Akt and FOXO1. This study aims to identify genes, gene interactions, and molecular pathways and processes associated with muscle aging and exercise in older adults that remained undiscovered until now leveraging on an artificial intelligence approach called artificial neural network inference (ANNi).

**Methods:**

Four datasets reporting the profile of muscle transcriptome obtained by RNA‐seq of young (21–43 years) and older adults (63–79 years) were selected and retrieved from the Gene Expression Omnibus (GEO) data repository. Two datasets contained the transcriptome profiles associated to muscle aging and two the transcriptome linked to resistant exercise in older adults, the latter before and after 6 months of exercise training. Each dataset was individually analysed by ANNi based on a swarm neural network approach integrated into a deep learning model (Intelligent Omics). This allowed us to identify top 200 genes influencing (drivers) or being influenced (targets) by aging or exercise and the strongest interactions between such genes. Downstream gene ontology (GO) analysis of these 200 genes was performed using Metacore (Clarivate™) and the open‐source software, Metascape. To confirm the differential expression of the genes showing the strongest interactions, real‐time quantitative PCR (RT‐qPCR) was employed on human muscle biopsies obtained from eight young (25 ± 4 years) and eight older men (78 ± 7.6 years), partaking in a 6‐month resistance exercise training programme.

**Results:**

*CHAD*, *ZDBF2*, *USP54*, and *JAK2* were identified as the genes with the strongest interactions predicting aging, while *SCFD1*, *KDM5D*, *EIF4A2*, and *NIPAL3* were the main interacting genes associated with long‐term exercise in older adults. RT‐qPCR confirmed significant upregulation of *USP54* (*P* = 0.005), *CHAD* (*P* = 0.03), and *ZDBF2* (*P* = 0.008) in the aging muscle, while exercise‐related genes were not differentially expressed (*EIF4A2 P* = 0.99, *NIPAL3 P* = 0.94, *SCFD1 P* = 0.94, and *KDM5D P* = 0.64). GO analysis related to skeletal muscle aging suggests enrichment of pathways linked to bone development (adj *P*‐value 0.006), immune response (adj *P*‐value <0.001), and apoptosis (adj *P*‐value 0.01). In older exercising adults, these were ECM remodelling (adj *P*‐value <0.001), protein folding (adj *P*‐value <0.001), and proteolysis (adj *P*‐value <0.001).

**Conclusions:**

Using ANNi and RT‐qPCR, we identified three strongly interacting genes predicting muscle aging, *ZDBF2*, *USP54*, and *CHAD*. These findings can help to inform the design of nonpharmacological and pharmacological interventions that prevent or mitigate sarcopenia.

## Introduction

Aging is accompanied by sarcopenia, defined as a gradual loss in lean skeletal muscle mass, strength, and endurance.^1^, ^2^ This condition correlates with increased risk of developing physical disabilities and a myriad of diseases including chronic obstructive pulmonary disease and death, making it a major public health concern.^3^, ^4^ Sarcopenia is a complex condition marked by increased catabolic activity with consequential loss of muscle mass, systemic inflammation, and elevated oxidative stress.^5^, ^6^ A greater understanding of the molecular mechanisms regulating muscle mass is urgent towards the prevention and treatment of this condition, co‐morbidities, and dependency among older people.^7^

As mentioned, sarcopenia is characterized by increased catabolic activity, leading to muscle loss and subsequent atrophy. Molecular pathways underpinning this catabolic activity include inactivation of Akt (protein kinase B), which causes the translocation of FOXO1 (Forkhead box protein O1) to the nucleus, leading to the expression of atrophy‐associated ubiquitin, the E3 ligases FBXO32 (F‐box protein 32), and TRIM63 (tripartite motif containing 63).^8^ An alternative catabolic pathway independent of Akt is TNF‐α (tumour necrosis factor alpha) activation, which regulates the downstream transcription factor NF‐κB (nuclear factor kappa B) that in turn leads to the upregulation of TRIM63.^9^ In addition, muscle aging is associated with increased expression of inflammatory cytokines such as TNF‐α and IL‐6 (interleukin 6), which stimulate muscle atrophy through the ubiquitin–proteasome pathway.^10^

Physical exercise is a nonpharmacological intervention and the only recommended treatment for sarcopenia patients, showing additional benefits in improving life expectancy and delaying the onset of age‐associated disorders such as osteoporosis, diabetes, atherosclerosis, and cardiovascular diseases.^11^, ^12^ Resistance training in particular has been shown to activate muscle protein synthesis, which induces an increase in muscle size and strength. Such increase is particularly relevant as it helps counteracting increased catabolic activity associated with muscle aging and to preserve muscle mass.^13^ It is generally recognized that resistance training increases muscle protein synthesis via phosphorylation of mammalian target of rapamycin (mTOR) through insulin‐like growth factor (IGF‐1) and Akt, which further leads to activation of p70S6K (70 kDa ribosomal S6 protein kinase).^14^ Although mTOR and inflammatory pathways play an important role in muscle aging and adaptation to exercise, other genes and pathways are likely to be involved given the complexity and scale of such events. An example is apelin, a small, secreted peptide hormone recently identified as a key molecular driver of muscle regeneration that decreases during muscle aging. For example, BGE‐105, an oral agonist of the apelin receptor APJ, has revealed positive effects in reducing symptoms of frailty and decreasing muscle loss.^15^, ^16^

Deep learning (DL), as a subdiscipline of artificial intelligence, is a rapidly evolving field emerging across healthcare with promising integration to develop tools for diagnosis, prognosis, and treatment management.^17^ DL functions similarly to the human brain by using multi‐layered neural network algorithms to make predictions, which enables the solving of complex problems thanks to the exponential increase in the data within models.^18^ Artificial neural network (ANN) is a DL technique that has succeeded in discovering new genes that have the strongest influence on the regulation of sarcomas,^19^ developing a more precise classification model to predict hypertension^20^ and responsiveness to hepatitis C treatment^21^ and providing clinical scores for the assessment of Alzheimer's disease severity.^22^ ANN has never been used to investigate the transcriptome associated with aging muscle or exercise in older adults. Most studies employ conventional statistical computing (e.g., linear and multiple regression analysis, R and Python programming) to detect differentially regulated genes.^11^, ^23^, ^24^ In this study, we aim to identify age‐ and exercise‐associated genes and to predict the strongest interactions between them using ANN that could have remained undetected using conventional statistical computing. Findings are expected to better inform exercise interventions and help identifying therapeutic targets for pharmacological interventions aiming to mitigate sarcopenia, the latter still lacking.

## Materials and methods

### Publicly available datasets

Four datasets were selected from the studies, GSE8479, GSE9419, and GSE117525, publicly available at the Gene Expression Omnibus data repository (Home ‐ GEO ‐ NCBI (nih.gov)). The inclusion criteria were RNA‐seq data of skeletal muscle of young (32 ± 11 years) and older healthy adults (73.5 ± 10.5 years) on exercise training with a minimum duration of 6 months. All time‐points of isolated RNA from biopsies taken after 24 h of intervention were included, and samples taken immediately post exercise and up to 24 h were excluded to avoid detecting transient gene expression. Datasets with <10 000 gene transcripts were excluded for accurate assessment and comparison of datasets. In total, two datasets of young versus older adults and two datasets of long‐term resistance exercise were included in the analysis, all deposited and searchable prior to 2020.

Subject characteristics of GEO dataset obtained from GSE8479 study: Skeletal muscle samples from healthy older (*n* = 25, 65–84 years, 12 female and 13 male) were collected of which 14 completed 6‐month twice‐weekly full body progressive resistance exercise training programme (pre‐ vs. post‐exercise). The participants had thorough screening before admission to the study (telephone screening, medical evaluation including past history, consent from physician, and resting electrocardiogram before and after submaximal exercise test) and were chosen to be relatively active (walking, cycling, golfing, and tennis three or more times a week) to study the effects of aging per se. Exclusion criteria included hypertension, evidence of coronary heart disease, congestive heart failure, chronic obstructive pulmonary disease, diabetes mellitus, renal failure, smoking, and orthopaedic disability.

Subject characteristics of GEO dataset obtained from GSE9419 study: Prior starting the study, 10 healthy older men (63–79 years) completed an evaluation including an electrocardiogram, routine clinical blood and urine chemistries, and a written medical history. All subjects had clinically normal heart, liver, and kidney functions with no presence of diabetes mellitus.

Subject characteristics of GEO dataset obtained from GSE117525 study: Skeletal muscle samples were obtained from healthy older (*n* = 41, 64–75 years, 15 female and 26 male) adults before and after 6‐month full body strength training programme three times per week. Before inclusion, medical history of all subjects was evaluated, and an oral glucose tolerance test and electrocardiogram before and after exercise test were performed. Exclusion criteria included cardiac and peripheral vascular disease, orthopaedic limitations, and type 2 diabetes. All subjects were recreationally active.

### Artificial neural network inference analysis

**Figure 1:**
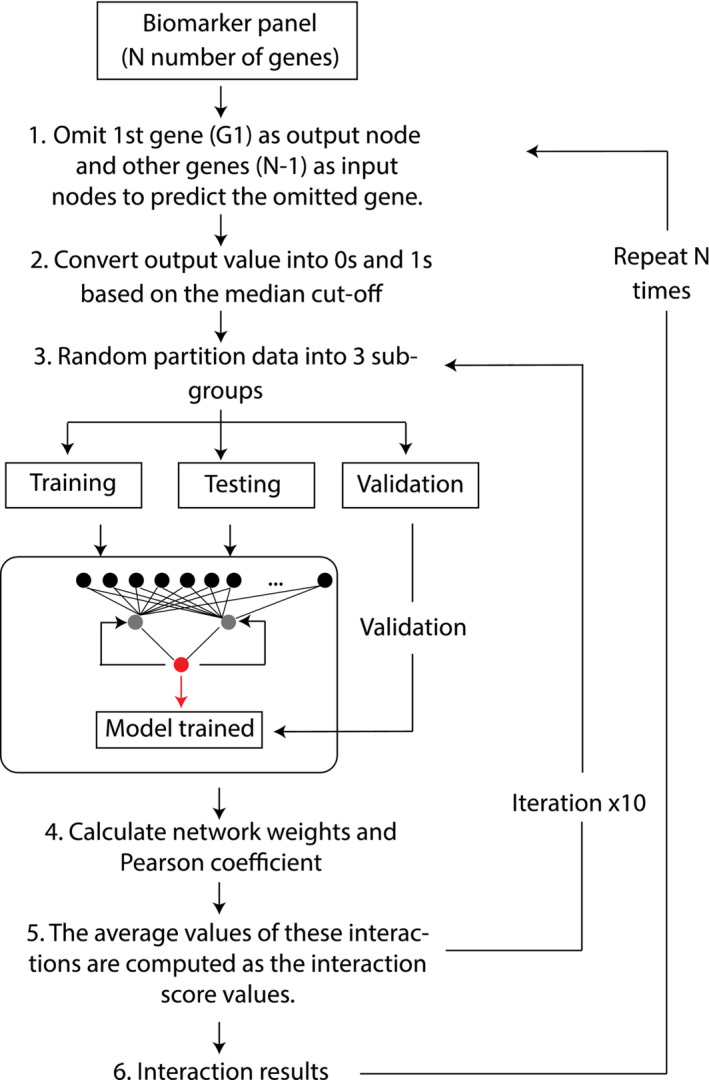
Overview of the interaction algorithm.

Enriched gene transcripts were fed into an artificial neural network inference (ANNi) algorithm (Intelligent Omics) to identify the top driver and target genes in aging and exercise (see Figure 1 representing schematics of the interaction algorithm). This approach utilized a swarm of smaller neural networks addressing part of a potential inferred network. This approach was selected over a single deep network that would lack transparency, ‘explainability’, and have (we have previously found) significant levels of redundancy and overfitting. The networks were trained to maximize generality using early stopping and regularization. The swarm of trained models were then parameterized and integrated into a deep network by integration of weights.

More specifically, multiple shallow neural networks were used to model the potential interaction between genes across multiple stochastically derived data sets. The underlying structure of the neural network is a weighted, directed graph, interconnecting artificial neurons (i.e., nodes) organized in layers with artificial synapses (i.e., links) that carry a value (i.e., weight), transmitting data (i.e., signals) from one node to the other nodes. All incoming signals from the input layer were processed based upon a set of defined parameters (i.e., error computation function, acceleration measure, and input weights) by the nodes in the intermediate layer (i.e., hidden layer), and an activation function was applied to the resulting sum. This sum was then used to determine the output result (i.e., predicted value) generated by the nodes in the output layer. This approach was then repeated across the ensemble of stochastically derived networks. Due to the connectionist computation in ANNs, the architecture of the ANN can be easily modified to address different questions and can compose complex hypotheses that can explain a high degree of correlation between features without any prior information from the datasets. Hence, a backpropagation MLP was chosen as the ANN to model the gene–gene interaction in this paper. The principle of the algorithm is to show the relationship between genes from the same pool, to shed light on how these molecules interact with each other and to identify new relationships between these molecules by iteratively calculating the influence that multiple variables may have upon a single one. The prediction weights and signal directions were used to model the strengths of the interaction signals and the direction of the interaction link between genes. The ANN model was validated using Monte Carlo cross‐validation to minimize the risk of overfitting and to optimize the generalization ability of the model.

### Interactome and pathway enrichment analysis

The top 200 driver and target genes previously identified by ANNi were used to generate interactome maps. To generate these maps, these genes were fed into the Cytoscape software with the STRING add‐on. To identify significantly enriched GO processes, signalling pathways and network processes, these same 200 top genes were fed into the MetaCore (Clarivate™) and Metascape databases.

### Study design and subject characteristics of muscle biopsies used for real time‐quantitative polymeraze chain reaction analysis

The most influenced and influential genes previously identified by ANNi were further investigated by RT‐qPCR of human muscle biopsies. Muscle biopsies were obtained from eight young (25 ± 4 years, 184 ± 6 cm and 83 ± 13 kg) recreationally active (skiing or team sports two times per week) males^25^ and eight older male adults (78 ± 7.6 years, BMI 25.4 ± 2.3).^26^ Aged participants completed a 6‐month resistance training programme with three sessions per week with 100% compliance. Assessment of oral glucose tolerance test and insulin sensitivity as well as investigation for cardiovascular disease, drug treatment, heart rate, blood pressure, body mass index, and smoking were performed. Hand grip strength, leisure‐time physical activity (four questions), and gait speed were measured prior initiation of the intervention. Biopsies from *m. quadriceps* were obtained before the training programme (baseline) and 24 h after the last training session (for aged adults). For analysis, comparisons were made between young and aged baseline and aged baseline versus aged exercised. Subjects were informed of the potential risks and discomforts prior to signing an informed consent form. All participants provided informed written consent. The study was approved by the Ethical Regional Board in Uppsala (Dnr 2012/154) and Ethical Regional Board in Umeå (2010‐223‐31M) and conducted in accordance with the Declaration of Helsinki.

### RNA isolation, reverse transcription, andreal time‐quantitative polymerase chain reaction

To assess the differential expression of the top drivers and target genes, RT–qPCR was employed, using to that end, muscle biopsies from eight young and eight aged male donors (see participant and intervention details in the previous section, Tables S2 and S3). One aliquot of ~10 mg frozen muscle tissue was homogenized using TRIzol (Invitrogen Life Technologies Carlsbad, CA), and total RNA was extracted. RNA concentration and purity were obtained by UV spectroscopy (Nanodrop 2000, Thermo Scientific). Three hundred nanograms of total mRNA was subsequently reverse transcribed into cDNA using an iScript™ cDNA Synthesis Kit (Bio‐Rad) in a total volume of 10 μL. Real‐time PCR was performed on a CFX96 Touch Real‐Time PCR Detection System (Bio‐Rad). The reaction mix consisted of 5 ng of the diluted cDNA template, 2× SYBR Green PCR Mastermix (Bio‐Rad), and 400 nM gene‐specific primers. The cycling procedures were 20 s at 95°C and 1 min at 95°C followed by 40 cycles at 95°C for 20 s and 60°C for 1 min. Primers were purchased from Sigma Aldrich. A complete list of primers used for RT–qPCR, including names and sequences, is provided in Table S1. Each individual sample was assayed on the same plate. GAPDH (Hs00172113_m1) was used as the housekeeping gene. For further control, b‐actin (Hs01375212_g1) was analysed as an additional reference gene. The results were almost identical with b‐actin or GAPDH as housekeeping genes. Hence, the GAPDH/β‐actin ratio did not change across time points. Target gene expression was subsequently reported as a ratio relative to the respective reference genes by the 2^−ΔΔCT^ formula.

Genes identified by ANNi as most influenced or influential but with no differential expression as per qPCR were interrogated using the MetaMEx database.

### Statistical analysis

The data were generally presented as mean ± the standard error of the mean (SEM). Statistical analysis was conducted using Graph Pad Prism for Mac Version 9.04. Relative gene expressions were compared using two‐sided unpaired (young vs. aged) or paired (before and after exercise in aged) *t* test with Welch's correction. *P* values <0.05 were considered statistically significant and were indicated within figures as *P < 0.05.

## Results

### Artificial neural network inference analysis identifies the genes *USP54*, *JAK2*, *CHAD*, and *ZDBF2* with the strongest interactions in skeletal muscle aging

**Figure 2:**
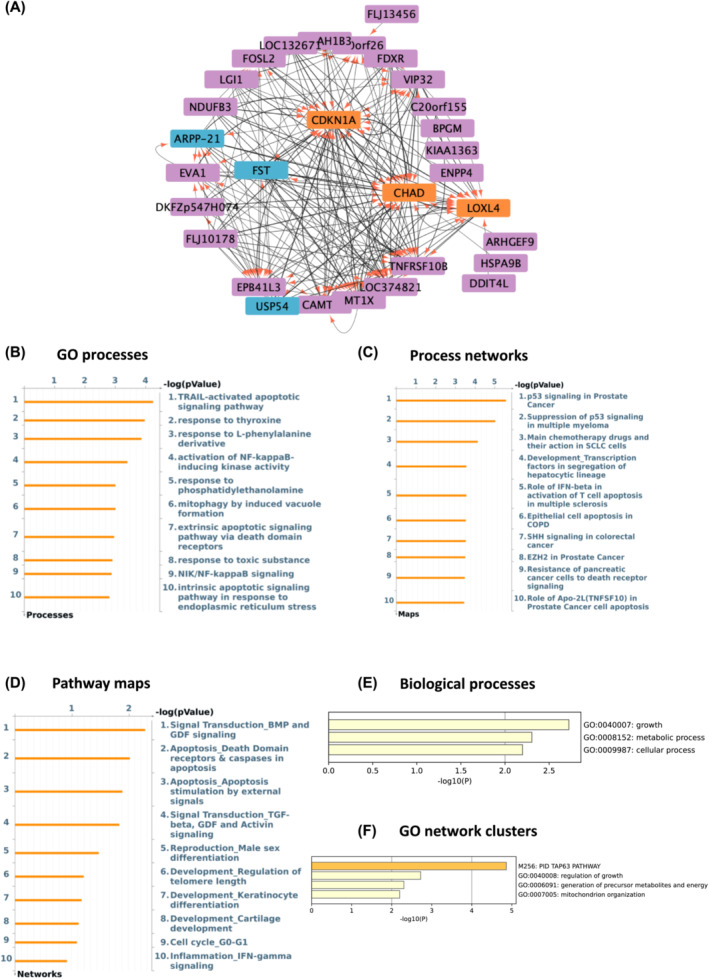
*USP54* and *FST* are the main drivers and *CHAD* and *CDKN1A* the main targets in aged muscle based on GSE8479 dataset. (A) Thickness represents the interaction strength, arrows the directionality, the most influencing genes (i.e., drivers) are in blue and the main influencers (i.e., targets) are depicted as orange in the interactome map. (B–D) Charts depicting top 10 overrepresented gene ontology GO cellular processes (B), process networks (C), and pathway maps (D) in aged versus young adults. (E, F) Metascape was used to create bar graphs indicating most significantly enriched biological processes (E) and networks clusters of GO terms (F) in age responsive genes with the strongest interactions as a result of ANNI analysis.

**Figure 3:**
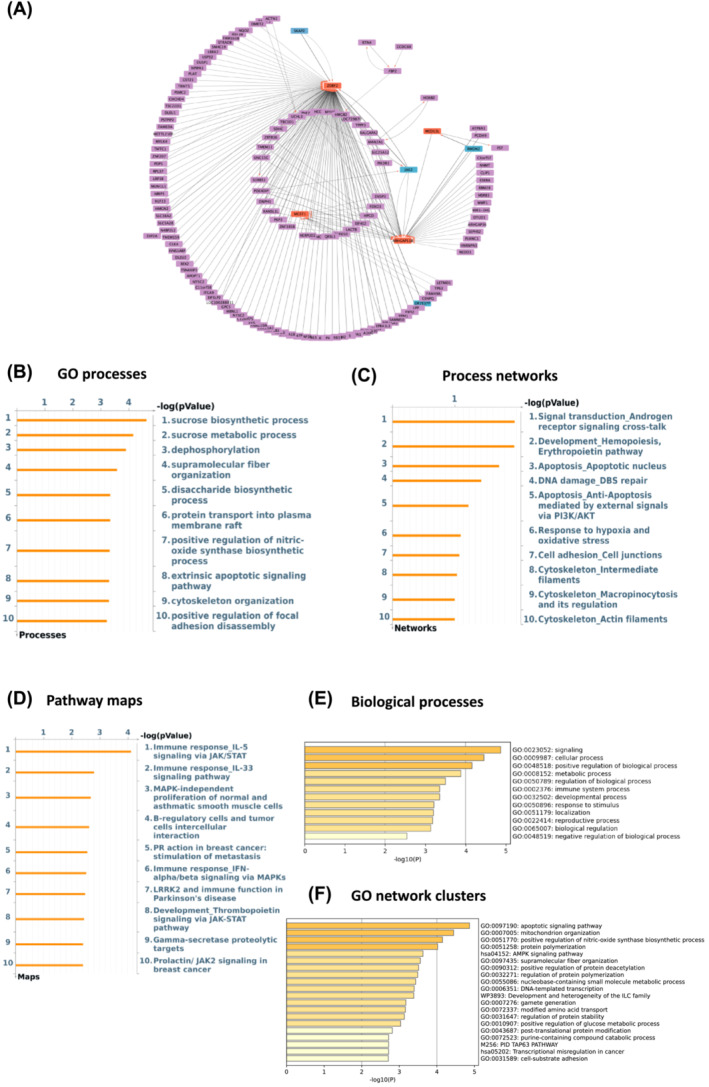
*JAK2* and *SKAP2* are the main drivers and *ZDBF2* and *ARHGAP11B* are top targets in aged muscle based on GSE9419 dataset. (A) Thickness represents the interaction strength, arrows the directionality, the most influencing genes (i.e., drivers) are in blue and the main influencers (i.e., targets) are depicted as orange in the interactome map. (B–D) Charts generated by Metacore depicting top 10 overrepresented gene ontology GO cellular processes (B), process networks (C), and pathway maps (D) in aged versus young adults. (E, F) Metascape was used to create bar graphs indicating most significantly enriched biological processes (E) and networks clusters of GO terms (F) in age responsive genes with the strongest interactions as a result of ANNI analysis.

ANNi analysis of differentially expressed transcripts allowed us to detect the most influential (drivers) and influenced genes (targets) associated with muscle aging. We identified *USP54* (Ubiquitin Specific Peptidase 54), *JAK2* (Janus Kinase 2), *FST* (Follistatin), and *SKAP2* (Src Kinase Associated Phosphoprotein 2) as the main drivers and *CHAD* (Chondroadherin), *ZDBF2* (Zinc Finger DBF‐Type Containing 2), *CDKN1A* (Cyclin Dependent Kinase Inhibitor 1A), and *ARHGAP11B* (Rho GTPase Activating Protein 11B) as the main targets. The interactomes created by Cytoscape software showing the top 200 drivers and targets with centralization and weighted interactions are depicted in Figures 2A and 3A.

Next, we investigated the GO processes, process networks and pathway maps associated with these same top 200 genes using Metacore. The most enriched GO processes were the TRAIL‐activated apoptotic signalling pathway and sucrose processing pathways (Figures 2B and 3B). With regard to process networks, p53 signalling and androgen receptor signalling were identified as the most significant ones (Figures 2C and 3C), while BMP‐GDF (adj *P*‐value 0.006) and IL5 signalling via JAK/STAT (adj *P*‐value <0.001) were found as the main pathway maps (Figures 2D and 3D). Interestingly, the extrinsic apoptotic signalling pathway was found among the top 10 GO processes in both age‐related datasets (adj *P*‐value 0.01, Figures 2B and 3B). Then, we used Metascape^27^ to identify the most enriched biological process and GO network cluster. The top‐level enriched biological processes were growth, signalling, metabolic, and cellular processes (Figures 2E and 3E). Regulation of growth, apoptotic signalling pathways, and mitochondrion organization were found to be the most enriched GO networks in the aging‐predicting datasets (Figures 2F and 3F).

### Artificial neural network inference analysis identifies the genes *EIF4A2*, *NIPAL3*, *SCFD1*, and *KDM5D* with the strongest interactions in response to exercise

**Figure 4:**
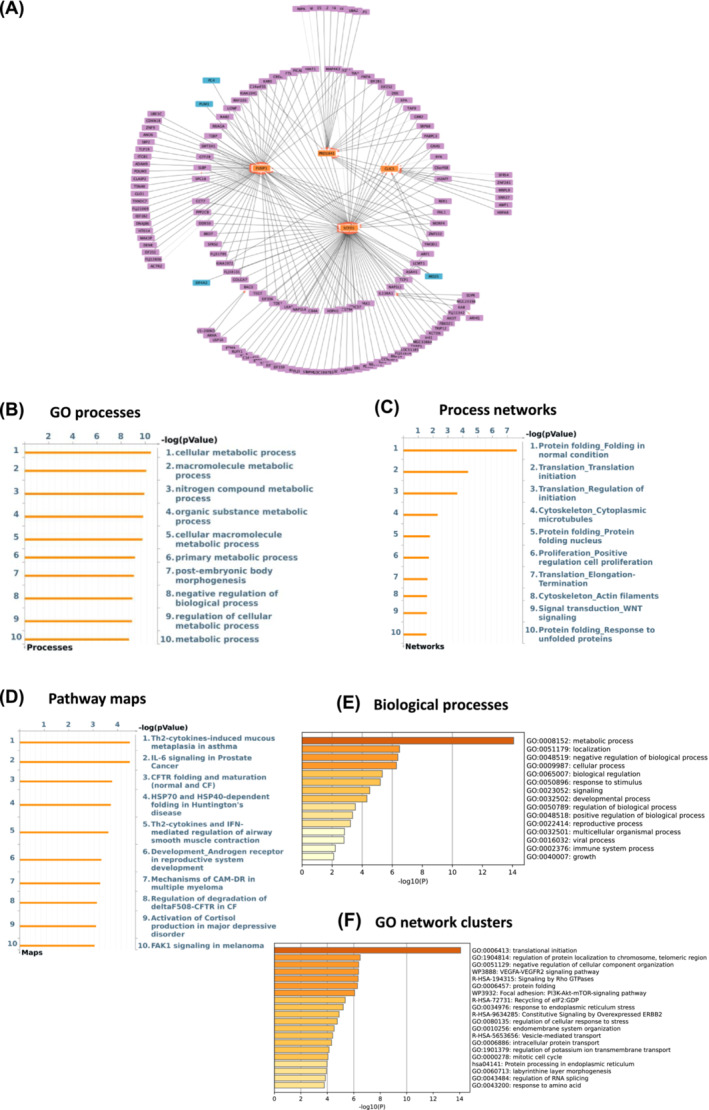
*EIF4A2* and *MO25* are the main drivers while *SCFD1* and *FUSIP1* are top targets in exercised aged muscle based on GSE8479 dataset. (A) Thickness represents the interaction strength, arrows the directionality, the most influencing genes (i.e., drivers) are in blue and the main influencers (i.e., targets) are depicted as orange in the interactome map. (B–D) Charts by Metacore depicting top 10 overrepresented GO cellular processes (B), process networks (C), and pathway maps (D) in response to exercise in older adults. (E, F) Metascape was used to create bar graphs indicating most significantly enriched biological processes (E) and networks clusters of GO terms (F) in exercise responsive genes with the strongest interactions as a result of ANNI analysis.

**Figure 5:**
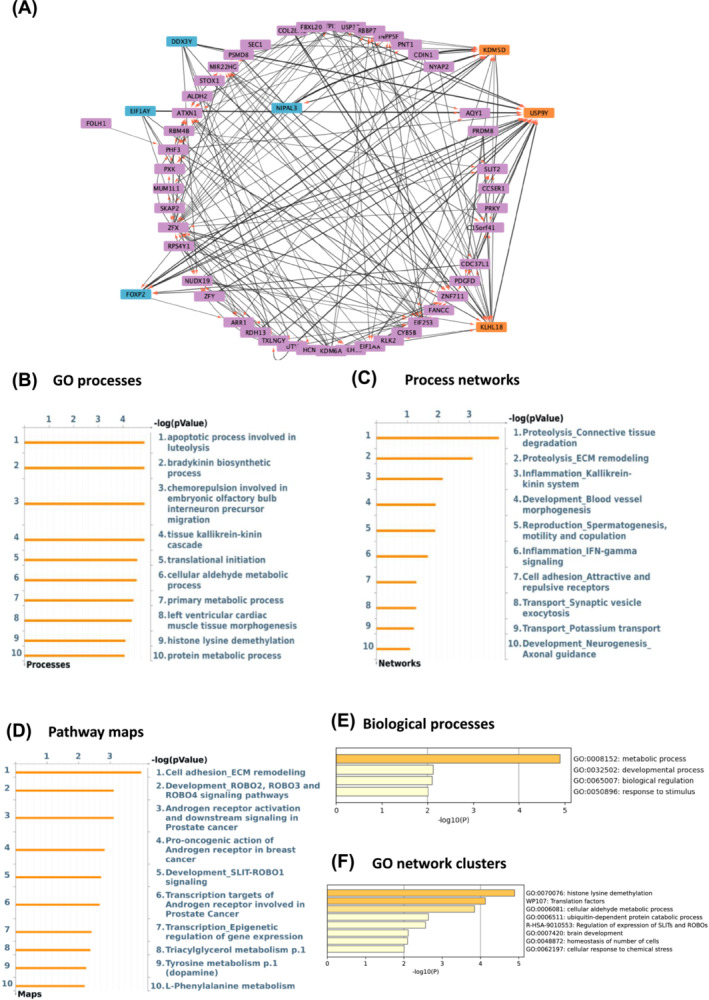
*NIPAL3* and *FOXP2* are the top drivers and *KDM5D* and *USP9Y* the main targets in exercised aged muscle based on GSE117525 dataset. (A) Thickness represents the interaction strength, arrows the directionality, the most influencing genes (i.e., drivers) are in blue and the main influencers (i.e., targets) are depicted as orange in the interactome map. (B–D) Metacore generated charts depicting top 10 overrepresented GO cellular processes (B), process networks (C), and pathway maps (D) in response to exercise in older adults. (E, F) Metascape was used to create bar graphs indicating most significantly enriched biological processes (E) and networks clusters of GO terms (F) in exercise responsive genes with the strongest interactions as a result of ANNI analysis.

ANNi analysis of differentially expressed gene transcripts was performed to identify the top drivers and targets associated with exercise. The top drivers among exercise‐responsive genes in the context of skeletal muscle aging were *EIF4A2* (Eukaryotic Translation Initiation Factor 4A2), *MO25* (Mouse protein‐25), *NIPAL3* (NIPA Like Domain Containing 3), and *FOXP2* (Forkhead Box P2) and the top targets *SCFD1* (Sec1 Family Domain Containing 1), *FUSIP1* (FUS‐interacting serine‐arginine‐rich protein), *KDM5D* (Lysine Demethylase 5D), and *USP9Y* (Ubiquitin Specific Peptidase 9 Y‐Linked), as depicted in Figures 4A and 5A, respectively. The most enriched GO processes were cellular and primary metabolic processes and apoptotic processes of luteolysis (Figures 4B and 5B), while protein folding (adj *P*‐value <0.001) and proteolysis (adj *P*‐value <0.001) were identified as the top process networks (Figures 4C and 5C). Regarding pathway maps, Th2‐cytokine‐induced mucous metaplasia in asthma and ECM remodelling (adj *P*‐value <0.001) prevailed among training studies (Figures 4D and 5D). Interestingly, process network of actin filaments within cytoskeleton was present in top for both aged and in exercised older skeletal muscle (Figures 3C and 4C).

Exercise‐responsive genes with the strongest interaction (top 200) that were fed into the Metascape database showed metabolic process, developmental process, and biological regulation as the most enriched GO biological processes (Figures 4E and 5E). Translational initiation, histone lysine demethylation, and translation factors were the top clusters with the most significantly enriched GO networks (Figures 4F and 5F).

### Real time‐quantitative polymeraze chain reaction confirmed significant differential expression of genes associated with muscle aging but not with exercise

**Figure 6:**
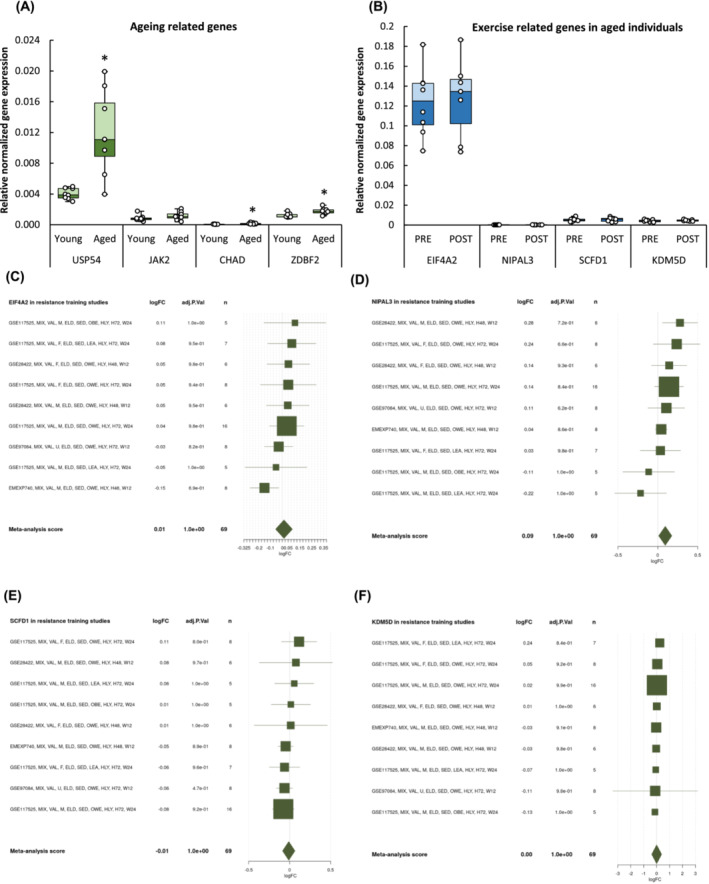
*USP54*, *CHAD*, and *ZDBF2* were significantly upregulated in aged muscle. (A) The differential expression of the most influenced (*CHAD* and *ZDBF2*) and influencing (*USP54*) age‐related genes were confirmed by real‐time PCR of young and aged muscle biopsies. (B) Exercise‐related genes in older adults remained unchanged. *P < 0.05 compared with young. (C–F) The online tool MetaMEx provides list of published studies on skeletal muscle response to exercise for a single gene with forest plots of individual statistics (fold‐change, FDR, 95% confidence interval) and meta‐analysis score.

Differential expression of the top drivers and targets genes associated with aging previously identified by ANNi was quantified by real‐time qPCR (Figure 6A). The genes *USP54* (*P* = 0.005), *CHAD* (*P* = 0.025), and *ZDBF2* (*P* = 0.008) were found to be significantly upregulated in aged individuals compared with young.

Similarly, differential expression of the top drivers and targets genes associated with resistance training previously identified by ANNi was quantified by real‐time qPCR (Figure 6B). These genes were not found differentially regulated in older individuals before and after exercise training (*EIF4A2 P* = 0.99, *NIPAL3 P* = 0.94, *SCFD1 P* = 0.94, and *KDM5D P* = 0.64). Next, we decided to interrogate whether these genes were previously associated with exercise using MetaMEx. After inputting *EIF4A2*, *NIPAL3*, *SCFD1*, and *KDM5D* into the MetaMEx database, and the search adjusted for healthy older subjects (>60 years) and filtered by 24‐week training duration, studies associated with resistance were found more than aerobic exercise training (Figure 6C–F). Taken together, these data suggest that *EIF4A2*, *NIPAL3*, *SCFD1*, and *KDM5D* can be associated with exercise in older adults.

## Discussion

ANNs have allowed the discovery of novel biomarkers of cancer^17^, ^19^ and are used in clinical practice to predict diseases such as Alzheimer's disease.^22^ In this study, we successfully employed it to discover novel genes related to muscle aging and resistance training, the interactions between them, as well as the most enriched GO processes and pathways. As we advance the current understanding of molecular events occurring in the aging muscle and during exercise, we are also promoting the design of more informed and targeted lifestyle interventions and pharmacological interventions aimed at mitigating sarcopenia.

Notably, ANNi analysis identified a set of strong interacting genes predicting muscle aging distinctive from what was previously detected in the original studies that employed traditional statistical computational methods. In the original studies the most significant genes were *FDXR* (ferrodoxin reductase), *UQCRH* (uniquinol‐cytochrome c reductase hinge protein), *SUCLA* (succinate CoA ligase), *UBA1* (ubiquitin‐activating enzyme E1), *CFLAR* (CASP8 and FADD‐like apoptosis regulator), and *SUMO3* (small ubiquitin like modifier 3).^28^, ^29^ In the present one, they are *CHAD*, *ZDBF2*, *USP54*, and *JAK2* with differential expression confirmed by RT‐qPCR for the first three genes.

*CHAD* is a member of leucine rich proteoglycan gene family, which is expressed in cartilage, as well as in bone, tendon, and human skeletal muscle.^30^ While *CHAD* deficient mice show altered cartilage and bone structural and functional development,^31^ downregulation of *CHAD* has been observed in exercised muscle transcripts,^32^ suggesting its involvement in skeletal muscle homeostasis as extracellular matrix component. *CHAD* expression levels were low in comparison with *ZDBF2* and *USP54* but still found to be significantly upregulated in aged muscle.

*ZDBF2* is known to be involved in DNA methylation during embryo development and genomic imprinting.^33^ This study is likely the first one identifying *ZDBF2* as one of the top target genes associated with skeletal muscle and/or aging.

*USP54* has a known function in the regulation of several tissues including skeletal muscle^34^ via ubiquitin–proteasome‐dependent proteolysis, which is a major protein degradation system with a crucial role in skeletal muscle homeostasis^35^ and therefore muscle aging. Significant upregulation of *USP54* gene expression in older *versus* young adults identified by ANNi and confirmed by RT–qPCR analysis corroborates the role of USP machinery in muscle atrophy.

*JAK2* mediates several signalling actions in cell growth, development and differentiation and is also associated with cytokine receptors.^36^ It was also associated with sepsis‐induced muscle wasting.^37^

GO analysis performed downstream to ANNI using Metacore suggests a significant enrichment of network processes and pathways maps related to apoptosis, cell signalling, and immune response. Previous transcriptome studies on muscle aging have reported similar findings—the enrichment of inflammation‐, apoptosis‐, and mitochondria‐related pathways,^7^, ^24^ suggesting a unique transcriptome signature of aging muscle.

Similar to aging, ANNi predicted exercise‐related genes quite distinctive from those previously identified using traditional statistical computational methods. Our analysis suggests that *SCFD1*, *KDM5D*, *EIF4A2*, and *NIPAL3* were the genes with the strongest interactions within the network in response to long‐term exercise in older adults while the original studies reported *SLP1* (secretory leukocyte peptidase inhibitor), *DDX223* (DEAD‐box helicase 23), and *CDKN1A* (cyclin dependent kinase inhibitor 1A) as the most significant. However, ANNi predicted genes were not differentially expressed between aged and young individuals. This is not entirely unexpected, because ANNi predicts gene interactions and their strength not the extent of differential gene regulation. So, although these genes were not differentially regulated, they can still to play a role in exercise in older adults due to the strength of their interactions with other genes. Further experiments including gene knockout models are necessary to validate the influence of *SCFD1*, *KDM5D*, *EIF4A2*, and *NIPAL3* in resistance training.

Bioinformatic analysis carried out downstream to ANNI using Metacore suggests the enrichment of process network and pathway maps related with ECM‐remodelling, protein folding, and cancer, whereas Metascape suggests dominance of biological process involved metabolism and development corroborating the notion that exercise can impact the muscle transcriptome.^38^ The original transcriptome studies suggest significant enrichment in genes related to mitochondrial regulation, extracellular matrix, glucose metabolism, and vascularization in response to chronic resistance training in older adults.^28^, ^39^

The *SCFD1* gene has been implicated in the pathogenesis of amyotrophic lateral sclerosis (ALS),^40^ while downregulation of *KDM5D* has been associated with poor prognosis in several tumours.^41^

*NIPAL3* is an integral component of the cell membrane predicted to be involved in magnesium ion transport^42^ while *EIF4F* has a crucial role in muscle cell differentiation via the PI3K/mTOR signalling pathway.^43^ Although no differential expression of *KDM5D*, *SCFD1*, *EIF4A2*, and *NIPAL3* in exercised aged muscle was confirmed by RT‐qPCR, after feeding the genes in the MetaMex software, these were confirmed to be previously associated with long‐term resistance training studies on older adults.

A question that imposes is whether the genes identified in this study by ANNi relate with genes well established in the literature to play a role on muscle aging including *AKT*, *FOXO1*, *FBXO32*, *TRIM63*, or *IL‐6*. A study showing reporting that IL‐6 induces muscle atrophy via gp/JAK2/STAT3 pathway suggests they do.^37^

To summarize, by applying ANNi analysis, we detected previously unreported muscle aging related genes, including *ZDBF2* and *CHAD*, and confirmed the role of *USP54*. Using GO analysis, we confirmed the involvement of signalling pathways and processes involving apoptosis, cell signalling, and immune response. This provides a valuable contribution to the field by not only improving our knowledge on aging muscle and the effects of long‐term exercise but also suggesting potential new targets for drug discovery and other therapeutic treatments aimed at ameliorating sarcopenia.

Future work will seek to undertake functional studies through siRNA knock out of these targets that will identify their potential role in phenotype in the aged and exercised muscle, which is lacking in the present study. Furthermore, future studies analysing datasets on sex differences dietary supplements and different training methods using ANNi would provide us with more information on biomarkers predicting aging and potential new therapeutic targets to combat sarcopenia.

## Conflict of interest

G.B. is the CSO for Intelligence Omics. The other authors declare no conflict of interests.

## Supporting information


**Table S1.** Primer sequences.
**Table S2.** Subject characteristics of aged muscle biopsy donors.
**Table S3.** Subject characteristics of young muscle biopsy donors.

## Data Availability

The publicly available skeletal muscle transcriptome data can be found in GSE8479, GSE9419, and GSE117525.
