# Supplementary material for: Artificial neural network inference analysis identified novel genes and gene interactions associated with skeletal muscle aging
Source: J Cachexia Sarcopenia Muscle. 2024 Aug 29;15(5):2143–55. doi: 10.1002/jcsm.13562 (PMC11446686; doi:10.1002/jcsm.13562)
Supplement: Supplementary file 1 — Table S1. Primer sequences. Table S2. Subject characteristics of aged muscle biopsy donors. Table S3. Subject characteristics of young muscle biopsy donors. [file JCSM-15-2143-s001.docx]

**Suppl Table 1. Primer sequences**

| Gene Name | Sequence 5’to 3’ | NCBI Gene ID |
| --- | --- | --- |
| NIPAL3 | Fw-TGTTCGTGTGCATGGTGGCAAC  Rv-CAACGGTGGTACGTGTGCTTGT | 57185 |
| SCFD1 | Fw- CAGAGCACAGGAAGATGAGGTC  Rv- CTGGAGTAGAAGGACACGAGAC | 23256 |
| ZDBF2 | Fw- ACAGGATGTACTGCAGCACCAC  Rv- CACCACGACGTCATGTAGGACA | 57683 |
| USP54 | Fw- GAGTTAGAGGCAGCGAAAGGGT  Rv- TGGGAAAGCGACGGAGATTGAG | 159195 |
| CHAD | Fw-CCTTTGGCAGATACCTGGAGAC Rv- CAGAGGTCCATAGACGGTTTCC | 1101 |
| EIF4A2 | Fw-CTCTCCTTCGTGGCATCTATGC Rv- CGTATCTACGGTGCTTCCTCTC | 1974 |
| JAK2 | Fw-CCAGATGGAAACTGTTCGCTCAG Rv- GACTCGCTTGTCAAAGGTAGACC | 3717 |
| KDM5D | Fw-GGCTGAGTCTTTTGACACCTGG Rv- GGTCCACAGTTTTCTGAGTCGG | 8284 |

**Suppl Table 2. Subject characteristics of aged muscle biopsy donors**

| **Sample ID** | **Age (y)** | **Sex** | **Height (cm)** | **Weight (kg)** | **Extension muscle torque (Nm)** | |
| --- | --- | --- | --- | --- | --- | --- |
| 02-025 | 80 | Male | 183 | 111.5 | 102.25 | 110.95 |
| 02-046 | 73 | Male | 183 | 96.1 | 194.05 | 253.3 |
| 02-114 | 71 | Male | 168 | 72 | 160.8 | 162.45 |
| 02-164 | 72 | Male | 179 | 115.3 | 267.2 | 264.1 |
| 02-174 | 79 | Male | 173 | 80.6 | 168.55 | 153.7 |
| 02-218 | 73 | Male | 172 | 78.3 | 150.1 | 153.65 |
| 02-221 | 84 | Male | 182 | 82.5 | 135.3 | 121.55 |
| 02-222 | 77 | Male | 174 | 100.5 | 180.1 | 171.85 |

**Suppl Table 3. Subject characteristics of young muscle biopsy donors**

| **Sample ID** | **Age (y)** | **Sex** | **Height (cm)** | **Weight (kg)** | **Isometric knee extension strength (Nm)** | | **Single leg incremental test to exhaustion (s)** | | **Mean fiber area (cm^2^)** | |
| --- | --- | --- | --- | --- | --- | --- | --- | --- | --- | --- |
|  | **PRE** | **PRE** | **PRE** | **PRE** | **PRE** | **POST** | **PRE** | **POST** | **PRE** | **POST** |
| A-1 Rest R | 21 | Male | 187 | 77.4 | 240.7 | 282.06 | 628 | 734 | 4941 | 5864 |
| A-2 Rest L | 27 | Male | 180.8 | 79.1 | 370.8 | 427 | 548 | 690 | 5415 | 4733 |
| A-3 Rest R | 26 | Male | 187.7 | 89.6 | 253.1 | 257.42 | 610 | 611 | 4970 | 4686 |
| A-4 Rest L | 22 | Male | 188.2 | 71.5 | 308.8 | 381.1 | 679 | 688 | 6774 | 5555 |
| A-5 Rest-R | 31 | Male | 177.2 | 97.6 | 246.9 | 265.16 | 528 | 754 | 5145 | 6362 |
| A-6 Rest L | 32 | Male | 191.3 | 80.2 | 302.9 | 387.8 | 370 | 720 | 2990 | 5133 |
| A-7 Rest R | 21 | Male | 178.4 | 86.3 | 228.3 | 210.28 | 614 | 730 | 3966 | 4240 |
| A-8 Rest L | 25 | Male | 192.2 | 109.2 | 328.1 | 340.04 | 626 | 720 | 4547 | 5353 |
